# The Distribution and Abundance of an Island Population of Koalas (*Phascolarctos cinereus*) in the Far North of Their Geographic Range

**DOI:** 10.1371/journal.pone.0059713

**Published:** 2013-03-18

**Authors:** Denise C. McGregor, Sarah E. Kerr, Andrew K. Krockenberger

**Affiliations:** Centre for Tropical Environmental and Sustainability Science, and School of Marine and Tropical Biology, James Cook University, Cairns, Queensland, Australia; Université de Sherbrooke, Canada

## Abstract

Koalas are an iconic species of charismatic megafauna, of substantial social and conservation significance. They are widely distributed, often at low densities, and individuals can be difficult to detect, making population surveys challenging and costly. Consequently, koala population estimates have been limited and the results inconsistent. The aims of this study were to estimate the distribution, relative abundance and population size of the koalas on Magnetic Island, far north Queensland. Population densities were estimated in 18 different vegetation types present on the island using a Fecal Standing Crop Method. Koala density ranged from 0.404 ha^−1^, recorded in forest red gum and bloodwood woodland, to absence from eight of the vegetation types surveyed. The second highest density of 0.297 koalas ha^−1^ was recorded in mixed eucalypt woodland, which covers 45% of the island. The total abundance of koalas on Magnetic Island, not including those present in urban areas, was estimated at 825±175 (SEM). The large variation in koala density across vegetation types reinforces the need for sampling stratification when calculating abundance over large areas, as uniformity of habitat quality cannot be assumed. In this context, koala populations also occur in low densities in areas generally regarded as poor quality koala habitat. These results highlight the importance of protecting vegetation communities not traditionally considered to have high conservation value to koalas, as these habitats may be essential for maintaining viable, widespread, low-density populations. The results from this study provide a baseline to assess future trends in koala distribution, density and abundance on Magnetic Island.

## Introduction

Koalas (*Phascolarctos cinereus*) are arboreal folivores that occur in the eucalypt forests of eastern Australia. Their current distribution is widespread, covering approximately one million square kilometers and 30 biogeographical regions, from the tropical forests of northern Queensland to the temperate forests of the Victoria coast [1], [2]. However, their distribution is not continuous, but patchy, composed of many separate populations isolated from other groups by unsuitable habitat [3]. This is primarily a reflection of forest fragmentation [4] as the distribution and density of koalas is limited by the presence of *Eucalyptus* and *Corymbia* species that comprise the koala diet [3], [5]. Due to their sedentary nature [3], koalas can be difficult to detect. This combination of factors makes estimating koala abundance difficult and costly [6]–[8], and has resulted in a limited number of estimates at regional, state and national levels [6], [9], [10].

The distribution of the koala has contracted by more than 50% from pre-European distribution [11], [12], with much of the reduction attributed to extensive fragmentation of koala habitat in Queensland [13]. The current distribution of koalas within their reduced range faces ongoing threats from clearing, fragmentation, expanding urbanization, disease, vehicular traffic, domestic dogs and bushfire [6], [12], [14], [15]. Koalas are also vulnerable to climatic extremes, particularly prolonged periods of unusually high temperatures and droughts [16], [17]. These conditions can lead to extensive leaf fall, subsequently affecting nutrient quality and moisture content available to koalas, resulting in population crashes [16]–[18]. As hotter and drier conditions continue, as is expected with climate change, koala populations will be adversely affected, with reduced populations restricted to diminishing riparian habitats [17]. This conclusion is supported by past observations of koala population crashes of as much as 80% in just 14 years associated with drought in southwestern Queensland [17], [18].

Comprehensive, reliable estimates of distribution and abundance are fundamental to the successful long-term conservation and management of a species [5], [19]. However, koala population studies have been performed predominantly in areas where koalas are known to occur in high densities [20]–[22], restricting the understanding of widespread, low density populations in many areas of northern, western and central Queensland [13], [23]. This increases the potential for inaccurate state and national population estimates. The extrapolation of broadscale koala density measurements from local studies is problematic in the absence of appropriate stratification as densities can range from 0.001 to 8.9 koalas ha^−1^ [20], [23]. Ignoring low-density populations of koalas over large areas can lead to underestimates [24]. This was the case on Kangaroo Island where the initial population estimate was calculated from surveys within 1400 hectares of high quality habitat while the remaining ∼200,000 hectares of medium and poor quality habitats were erroneously disregarded as having insignificant koala abundance [24]. This error resulted in a population size underestimate of 22,000 koalas [22], [24] and ultimately rendered the $1.25 million management scheme ineffective [22]. Many researchers agree that until additional, consistent and robust estimates of local, regional and national abundance can be made, and population trends clarified, inconsistencies will continue to hinder conservation efforts [6], [25], [9], [10].

### Survey Methods

Nationally recognized standards for the assessment of koala distribution and abundance have not been established. Abundance and density estimations have primarily been obtained from transect counts, with fixed boundaries [20], [23], [26]–[30]. Inference from community surveys [11], [14]; distance sampling [8]; mark-resight [19], [22], [31] and fecal pellet surveys [25], [17] have also been used. However, abundance surveys of low density, patchy koala populations, scattered over large regions, such as those typical of Queensland, have been challenging for conventional methods, such as direct counts, where even extensive efforts can lead to limited data or overlooked animals and inaccurate results [6].

Counting indirect animal signs (e.g., scat, nests, calls, tracks) provides an alternative to surveying elusive, low-density animals [32] that occupy dense or widespread habitats [33] such as the koala [34]. The Fecal Standing Crop Method (FSCM) [35], [36] can be used to calculate absolute abundance of animals from fecal pellet abundance estimated from transect searches [37]. This method requires pellet abundance to be divided by two additional parameters; the daily rate of pellet production of the species and the decomposition rate or maximum age of pellets collected from transects [34]. Koalas are highly suited to this method as the required parameters can be accurately estimated. Koalas have distinctive fecal pellets that are easily found under trees they have occupied [7], [38]. This contrasts sharply with the difficulty of locating these elusive animals [34]. While defecation rates are difficult to establish for most species, the sedentary nature and roosting behavior of koalas [3] facilitates this estimation [25].

Given that a large proportion of koala populations occur in Queensland [11], accurate estimations of their abundance within the state are an essential component to conservation management of the species [19]. Magnetic Island, a popular tourist destination in north Queensland, is economically and environmentally affected by the presence of a population of koalas introduced in the 1930's as a conservation response to population crashes and reductions in distribution throughout the koala's range [3], [16]. Unlike koala populations in isolates in the south of their range, there is no evidence that the Magnetic Island population has experienced extreme boom and bust cycles of population growth [3], [39], [40], [41]. As this population is located at the northern limit of the koalas' range it may represent a sentinel population for monitoring abundance trends under future climates. The aims of this study were to determine the distribution, relative abundance and population size of the koalas on Magnetic Island. This information had not previously been assessed and will be instrumental for future koala conservation and management and will provide future opportunities to monitor population trends in this closed population of koalas over time.

## Materials and Methods

### Ethics Statement

This study complies with the Australian National Health and Medical Research Council's Code of Practice for Care and Use of Animals for Scientific Purposes (2004) and Queensland State legislation and was approved by the James Cook University Animal Ethics Committee (A1343) and QLD NPWS Scientific Purposes permit WITK05490308.

### Magnetic Island Study Area and Climate

Magnetic Island is situated 5 kilometers off the north Queensland coast of Australia, near Townsville (19°08′S 148°50′E). The island is approximately 5184 hectares in area and includes Magnetic Island National Park (2716 hectares). The island's rugged peaks rise sharply from sea level to its summit, Mt Cook (497 meters above sea level). A plateau, divided by higher peaks with slopes exceeding 40°, occurs above 200 m [42]. The island is dominated by massive, exposed granite boulders. There is no permanent watercourse except a small tributary to Gustav Creek (pers. obs.). Urban settlements are restricted to the relatively flat areas of Horseshoe, Geoffrey, Nelly, Picnic, Bolger, and Young Bays. The human population is estimated at just over 2100 [43].

The climate of Magnetic Island is characterized by warm, dry winters and hot wet summers. July is usually the coldest month (mean daily minimum of 13.6°C) and December is typically the warmest month (mean daily maximum of 31.5°C) [44]. The wet season occurs from December to March and includes 75% of the 1196 mm average annual rainfall [44]. On average, September is the driest month of the year (mean monthly minimum rainfall of 10.8 mm) with February typically the wettest (mean maximum rainfall of 307.1 mm) [44]. Tropical cyclones occur in this area, with the most recent being cyclone ‘Yasi’ that struck Magnetic Island in January 2011, bringing torrential rain and destructive winds with gusts of 135 km/hr [45].

This study was conducted between August and October, 2011, during the dry season. During this period, the monthly mean maximum temperatures were 26.0°C for August, 27.7°C for September and 29.4°C for October and the monthly mean minimum temperatures were 14.7°C for August, 17.4°C for September and 20.7°C for October [44]. The total monthly rainfall on Magnetic Island for the same period was recorded at 0 mm in July, August and September and 15.8 mm in October [44]. Rainfall in October primarily occurred on two days; 3.4 mm on October 17 and 11.2 mm on October 15 [44].

### Vegetation Patterns and stratification of study sites

Geology, landforms and soil patterns were used by Sandercoe [42] to categorize Magnetic Island into five landform classifications with differing vegetation communities: I) Foreshore unconsolidated sediments, II) Coastal lowlands and sands and piedmont deposits, III) Granite hills and Lithosols and talus slopes, IV) Plateau and hills of Mt Cook, V) Agglomerate hills of the West Point area. These five landforms were further divided into 23 vegetation types (Table 1) [42].

**Table 1:** Vegetation types of Magnetic Island grouped by major landform divisions.

Magnetic Island Vegetation Types			Area (hectares)	Area (% of island)
**I**	**Type**	**Foreshore unconsolidated sediments**		
	1.	Stilted mangrove forest	98.2	2.0
	2.	Grey mangrove forest	2.9	0.6
	3.	Mixed mangrove shrubland	82.3	1.6
	4.	Saltmarsh and samphire flats	51.9	1.0
**II**		**Coastal lowlands on sands and piedmont**		
	5.	Coastal sheoak woodland	27.9	0.6
	6.	Weeping teatree and bulkuru swamp	19.9	0.4
	7.	Moreton bay ash flats	182.2	3.7
	8.	Forest red gum and bloodwood woodland	25.4	0.5
	9.	Poplar gum and bloodwood woodland	31.6	0.6
	10.	Littoral scrub	34.6	0.7
**III**		**Granite hills of lithosois and talus slopes**		
	11.	Araucaria forest	42.0	0.8
	12.	Mixed lowland coastal forests	127.5	2.6
	13.	Low vine forest amongst boulders	263.0	5.3
	14.	Vine forest	94.3	1.9
	15.	Mixed semi-deciduous woodland	319.4	6.4
	16.	Mixed semi-deciduous low open woodland	116.1	2.3
	17.	Mixed eucalypt woodland	2232.9	44.8
	18.	Acacia scrubland	152.8	3.1
	19.	Grassland +/− sparse trees and scrubs	197.6	4.0
	20.	Mallee brush box forests	145.5	2.9
**IV**		**Plateau and hills of Mt. Cook**		
	21.	Cabbage tree palm and forest sheoak forest	73.8	1.5
	22.	Forest sheoak and grass tree shrubland	31.9	0.6
**V**		**Agglomerate hills of the West Point area**		
	23.	Mixed open low scrub	87.26	1.7
	24.	Disturbed (urban) areas	548.43	11.0

(Adapted from Sandercoe 1990).

Eighteen of the 23 vegetation types contain at least one of the 15 *Eucalyptus* or *Corymbia* species found on the island [42]. Mixed eucalypt forest (vegetation type 17) covers 45% of the island and contains the greatest diversity of eucalypts with 12 species [42]. The most predominant species are yellow stringybark (*Eucalyptus acmenoides*), narrow-leafed ironbark (*Eucalyptus drepanophylla*), pink bloodwood (*Corymbia intermedia*), ghost gum (*Corymbia aparrerinja*), and Carbeen (*Eucalyptus tessellaris*) [42]. Koalas in the mixed eucalypt forest of Magnetic Island have previously been found to prefer *Corymbia intermedia*, *Corymbia erythrophloia* and *Eucalyptus drepanophylla* (Tindall pers. comm.).

Koala densities can vary significantly between differing habitats [22], [23] so the survey was stratified using the 23 vegetation types identified by Sandercoe [42]. Vegetation areas 1–5 were not surveyed as they were classified as mangroves, saltmarshes, samphire flats and sand dunes. Eucalypts do not occur in these vegetation types [42], nor had koala sightings been documented in these areas by local rangers (Petersen pers. comm.). Approximately 1% of the total area of each vegetation type was surveyed, or a minimum of six transects, whichever was greater. Transects were chosen from at least two areas from each vegetation type, except vegetation types that occurred only in area (vegetation types 9, 21 and 23). Areas within each vegetation type were selected for maximum size and distance from other surveyed areas of the same vegetation type. Urban areas were not surveyed as they are continuously disturbed by human activities such as supplemental watering, mowing, and gardening. These activities could result in the removal of pellets or an increase in their decay rate; compromising the accuracy of the FSCM.

### Fecal Standing Crop Method

**Figure 1:**
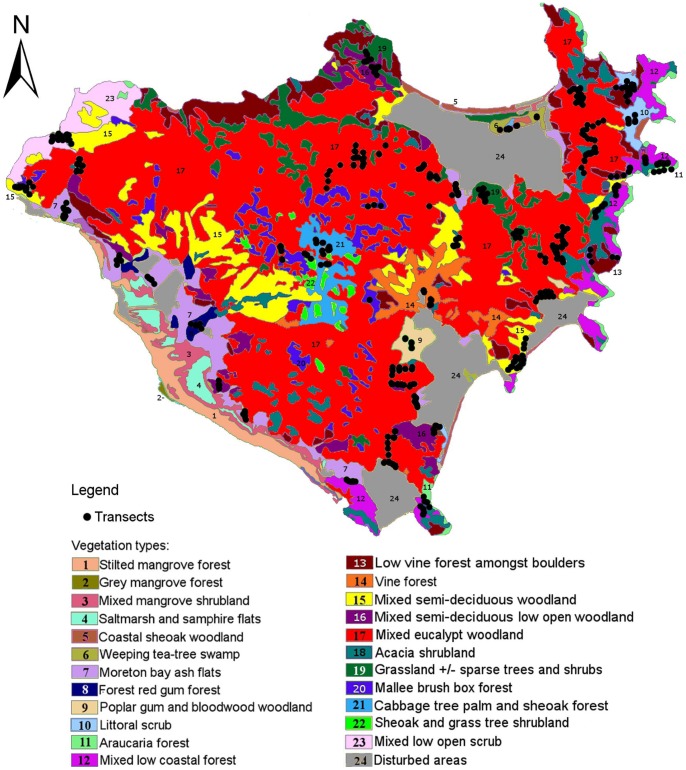
Map of Magnetic Island depicting vegetation types, urban areas and starting locations of transects searched. (Adapted from Sandercoe 1990).

Fecal pellet searches were conducted between August 31 and October 27, 2012, across a total of 385, 2×100 m strip transects (Fig. 1). The first transect in each survey area was established by locating a randomized starting point, with additional transects located systematically, with a distance of 10–20 m separating transects. A GPS (Garmin Etrex) was used to locate the designated random starting point in each area and subsequent start and finish points of each adjacent transect. Transect lines were walked by a single observer in one direction, who searched and collected fecal pellets. A GPS and compass were used to navigate the transect line while a one meter stick was used to measure the distance from the line. Substrate was thoroughly searched in each transect. All vertebrate fecal pellets found, including those deposited by koalas, wallabies, possums and rodents, were collected in sealable plastic bags. Each cluster of koala fecal pellets was placed into a separate plastic bag, while all non-koala fecal pellets were placed in one bag for each transect. All fecal pellets were assigned to species by DM at the end of each day. Search times per transect ranged from 45 minutes to three hours depending on the substrate type, density of vegetation and quantity of pellets to be collected. The total search time across all 385 transects was approximately 656 hours. The raw data from transect searches, including transect name, search date, start and end coordinates, transect area and number of pellets found is available online from the Tropical Data Hub at James Cook University.

Daily pellet production rates were estimated in 15 free-ranging koalas (7 adult females, 5 adult males and 3 subadults). Plastic sheeting was placed under the entire canopy of each tree with a koala present, and secured in place with rocks or fallen branches, to separate previously deposited pellets and assist in the collection of fresh pellets [45], [46]. Pellets deposited were counted as long as each koala remained up the tree, and the occupancy time was recorded in minutes. The minimum occupancy period was 11 hours and the maximum was 23 hours. Daily estimates were then calculated from the mean number of pellets produced per minute.

The maximum age of pellets in abundance counts was estimated using a method developed by Sullivan et al. [7] based on the volatile essential oil component of eucalypt leaves, the primary component of the koala diet [47], which steadily diminishes as pellets age [7]. Fecal pellets were categorized into age classes based on their level of eucalypt odor with ‘new’ pellets classified as those having any eucalypt odor and “old” pellets as having no odor. A single researcher (DM) determined the presence or absence of odor in all koala pellets collected from transect searches to control for variations in individual subjective sensitivity to detect odors. Color differences were noted between fresh pellets collected from various geographic areas on the island; therefore color was not used in determining age, as was done in other studies [7], [25].

Trials to determine the number of days for which koala fecal pellets maintained their eucalypt odor were conducted concurrently with fecal pellet searches in transects. Twenty groups of 70 fresh pellets were collected over five weeks, from August 29-September 30, from defecating koalas across a variety of locations within the study area. Pellets were then allowed to age in a leafy substrate under eucalypt trees in the study area and one pellet from each group was tested for odor each day until no internal eucalypt odor was detected for three consecutive days. In this way the maximum age of ‘new’ pellets was established.

Based on the parameters established for the daily pellet production rate and the maximum pellet age in transect searches, the FSCM [25], [35] was used to calculate the mean koala density (± SEM) in each of the 18 vegetation types surveyed. This density was then extrapolated over the total area occupied by each vegetation type to derive an estimated absolute abundance of koalas in each vegetation type and an island-wide population estimate.

## Results

Koala fecal pellets maintained their eucalypt odor for a mean of 57 days ±1 day, (SEM, n = 20). This estimate was used as the maximum age of ‘new’ pellets in the application of the FSCM. The mean daily production of koala fecal pellets for free-ranging koalas was 141±11 (95% CI, n = 15; Range 108–168 pellets). A total of 11,073 ‘new’ koala fecal pellets were collected from 138 of the 385 transects searched (36%). Other fecal pellets collected from transects included 8775 ‘old’ koala, 19,290 allied rock wallaby (Petrogale assimilis), 8799 brushtail possum (Trichosurus vulpecula), 4308 agile wallaby (Macropus agilis) and 747 unidentified rodent pellets.

**Figure 2:**
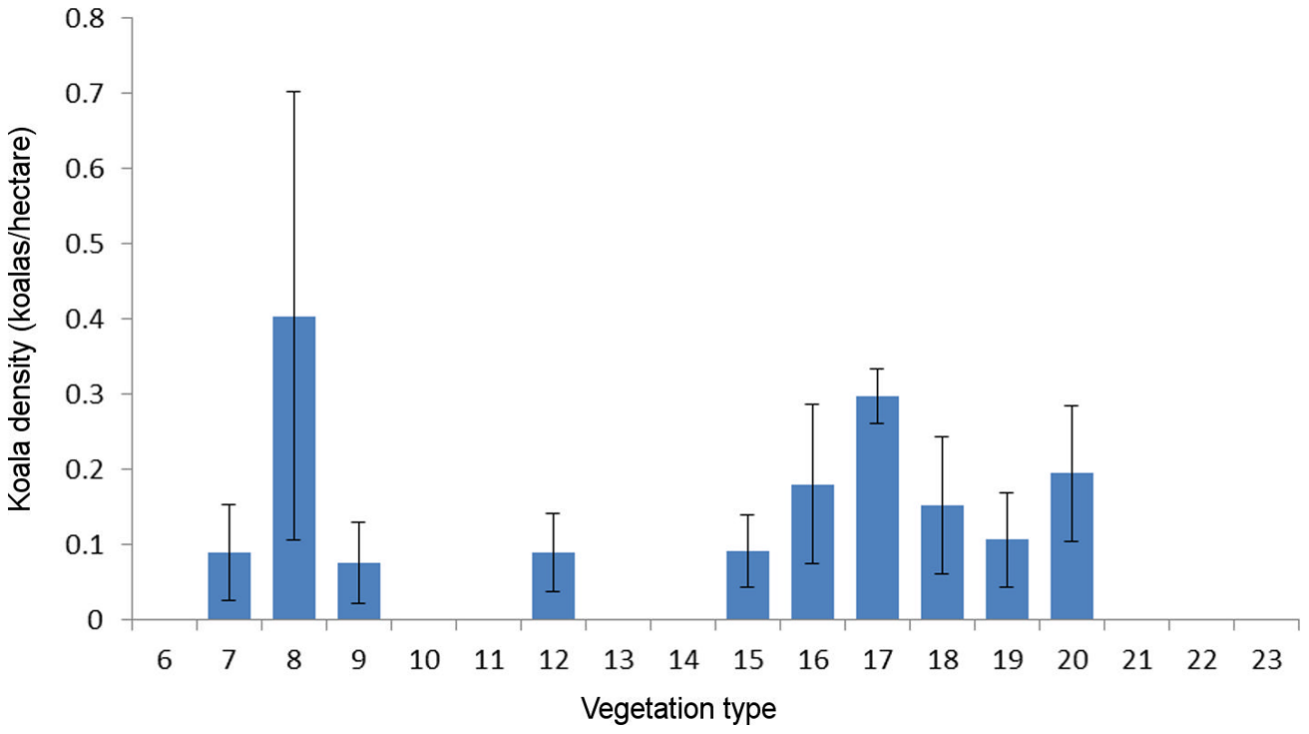
Density of koalas (koalas ha^−1^) across 18 vegetation types on Magnetic Island. The mean density of koalas ± SE are shown for each vegetation type. Density estimates were derived using the fecal standing crop method. The 18 vegetation types surveyed were classified as per Sandercoe (1990), and included: 6: weeping tea-tree swamp; 7: Moreton bay ash flats; 8: forest red gum forest; 9: poplar gum and bloodwood woodland; 10: littoral scrub; 11: aracaria forest; 12: mixed low coastal forest; 13: low vine forest amongst boulders, 14: vine forest; 15: mixed semi-deciduous woodland; 16: mixed semi-deciduous low open woodland; 17: mixed eucalypt woodland; 18: acacia shrubland; 19: grassland ± sparse trees and shrubs; 20: mallee brush box forest; 21: cabbage tree palm and sheoak forest; 22: sheoak and grass tree shrubland; 23: mixed low open scrub.

**Figure 3:**
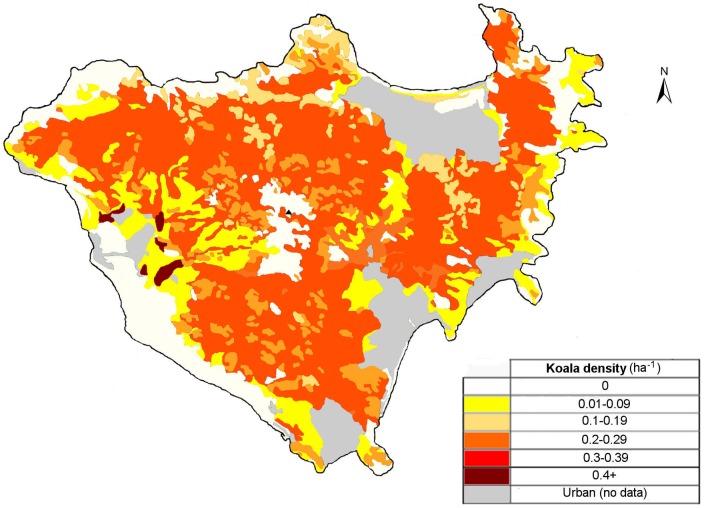
Map of Koala density (koalas ha^−1^) across Magnetic Island as determined using the fecal standing crop method, with stratification by vegetation type.

**Figure 4:**
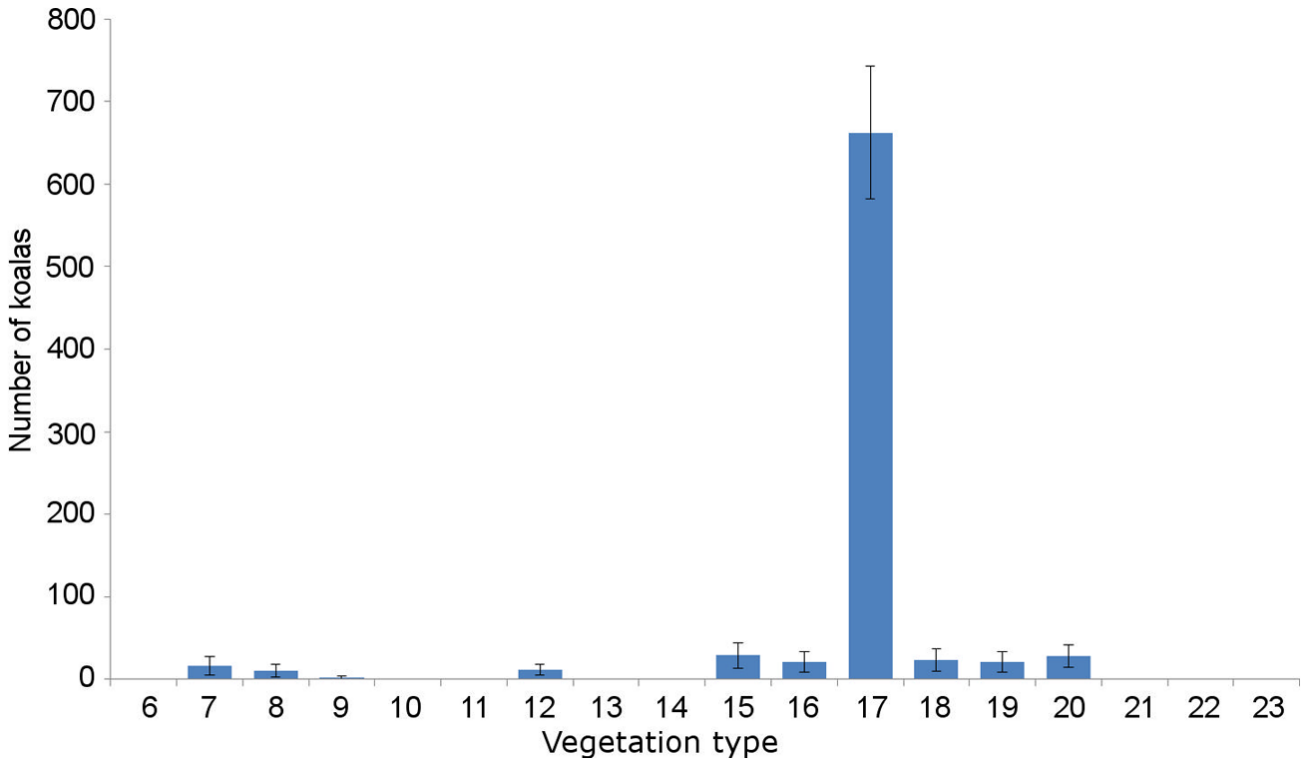
Abundance of koalas across 18 vegetation types on Magnetic Island. The mean abundance of koalas ± SE are shown for each vegetation type. Abundance estimates were derived using the fecal standing crop method. The 18 vegetation types surveyed were classified as per Sandercoe (1990), and included: 6: weeping tea-tree swamp; 7: Moreton bay ash flats; 8: forest red gum forest; 9: poplar gum and bloodwood woodland; 10: littoral scrub; 11: aracaria forest; 12: mixed low coastal forest; 13: low vine forest amongst boulders, 14: vine forest; 15: mixed semi-deciduous woodland; 16: mixed semi-deciduous low open woodland; 17: mixed eucalypt woodland; 18: acacia shrubland; 19: grassland ± sparse trees and shrubs; 20: mallee brush box forest; 21: cabbage tree palm and sheoak forest; 22: sheoak and grass tree shrubland; 23: mixed low open scrub.

Koala fecal pellets were found in 10 of the 18 surveyed vegetation types. These vegetation types contained significantly different densities of koala pellets (χ^2^ = 72.15, df  = 17, p = 0001). Koala density ranged from 0.404 ha^−1^ to zero koalas ha^−1^ (Fig. 2; Fig. 3). The highest density was recorded in forest red gum and bloodwood woodland (vegetation type 8). The total abundance of koalas on Magnetic Island was estimated at 825±175 (SEM; Fig. 4). There were no koala fecal pellets, and hence no koalas, in eight of the vegetation types surveyed. The vegetation type with the highest koala density covers only ∼25 hectares or 0.05% of Magnetic Island, so, despite the high density, only 1% of the island's koalas occurred there (Fig. 4). The second highest density of 0.297±0.036 koalas ha^−1^ was recorded in mixed eucalypt woodland (vegetation type 17), which includes 2233 hectares or 45% of the island and supports 80% of the island's koala population.

## Discussion

This study recorded koala densities of 0–0.40 ha^−1^ using the FSCM. These results fall within the range of koala densities from other Queensland area studies using various methods including the FSCM (0–2.51 koalas ha^−1^) [25]; distance sampling (0–0.76 koalas ha^−1^) [8]; and direct counts (0.1–2.0 koalas ha^−1^) [16], 0.4 koalas ha^−1^ [28], (0.02–0.4 koalas ha^−1^) [29]. Although the density from this study is broadly consistent with other studies from Queensland, it is much lower than abundances on southern islands [41]. For example, koala densities reach 6–8.9 koalas ha^−1^ on French Island [48], and 5 koalas ha^−1^ on Kangaroo Island [22]. These islands have a history of population spikes followed by overbrowsing, koala starvation and dramatic population crashes [41]. Despite similar numbers of koalas being introduced to those islands over a similar time period, koala density on Magnetic Island has remained considerably lower than southern islands, without documented population crashes. However, direct comparisons of density between these extreme limits of the koala range are difficult due to wide variations in habitats and the unknown sustainable carrying capacities on northern islands.

### Pellet age

Pellets 56.70±1 day old or less were classified as ‘new’ according to odor. This is considerably longer than the ‘new’ classification determined previously [7] and likely the result of the absence of rainfall during this study as well as differences in the aging method. Sullivan et al. [7] classified pellets with a strong eucalypt odor as ‘new’ and pellets with a weak eucalypt odor as old. In the current study, pellets were considered ‘new’ until there was complete absence of eucalypt odor. However, because the categorization was consistent within this study it is unlikely that this difference in methodology between studies resulted in any impact on the estimations of koala density.

Even within the same species, pellet aging has been shown to be highly variable based on differences in habitat and climatic events [7], [49], [50]. To minimize errors associated with pellet age, measurement of pellet aging should occur in the time period just prior to and concurrent to pellet sampling [25]. Longer decay rates in koala fecal pellets have been associated with dry climatic conditions [50], whereas increased rainfall and substrate moisture has been suggested to increase the breakdown and relocation of feces [32], [35]. Consequently, the dry season is the most appropriate survey period for implementing the FSCM in the tropics of Queensland.

We observed that throughout the nine weeks of aging trials, pellets did not show cracking or signs of disintegration. However, once past the critical ageing threshold, pellets quickly lost their eucalypt odor. The slow decay rate of koala pellets minimize the risk of underestimation but may increase the risk of overestimation if pellets cannot be aged accurately [32], [50]. For example, large amounts of ‘old’ pellets occur along dry creek beds where they appear to have accumulated during the wet season (pers. obs.). Counting only ‘new’ pellets, as opposed to aging pellets from defecation to decay, limited the variability of prolonged environmental and biotic factors on the breakdown and relocation of pellets.

### Pellet deposition rates

Defecation rates can be difficult to establish as it is essential to know the exact amount of time animals spent in the area and the amount of dung accumulated in that time [32]. One of the disadvantages commonly identified with the FSCM is the evasive movement of animals [51] that prevents accurate measurements of defecation. Consequently, for most animals, estimating this parameter requires confining them in a small area, often necessitating artificial feeding, which may alter defecation rates [52]. However, as koalas are sedentary by nature [3], defecation rates can be easily estimated. Our estimate of koala daily pellet production of 140.60±10.85 (95% CI) is consistent with the other two published estimates of 150.75±12.55, in free-ranging koalas, [25] and 174±29 pellets day in captive koalas [46].

Due to the low nutrient, sclerophyllous diet of koalas it has been suggested that their digestive processes are likely to be regular instead of episodic, and generally uniform across habitats [53]. However, Ellis et al. [45] found koala pellets deposited disproportionally over a diurnal cycle, with higher numbers of pellets at peak activity times from 1800–2400 hours and therefore suggested pellets be collected over a 24 hour period to avoid bias from potential circadian activity patterns. In this study, pellets were collected from 14 of the koalas for approximately 12 hours from 600–1800 hours (one koala remained in the tree for 23 hours). Sullivan et al. [25] collected pellets over a 24 hour period and reported a 9% higher mean daily pellet production rate in free-ranging koalas then we found. If we have underestimated defecation rates due to measurement largely during daylight hours, then applying the pellet production rate measured by Sullivan et al. [25] suggests our study could have overestimated the population by 9% (74±16 koalas), well within our estimate of error.

Sullivan et al. [25] found their upper estimates to underestimate koala density by <20% when compared with direct counts of koalas in the same area. To avoid possible underestimation of koala density in the current study, the entire area of each transect was thoroughly searched, as opposed to only under the canopies of eucalypt trees [17], [25]. While non-eucalypt trees contribute only a minor part of the koala diet, tree species not preferred for food, including non-eucalypt genera, are used opportunistically by koalas for roosting and sleeping [11], [54], [55]. Consequently, we do not expect the same overall underestimate in this study.

### Conservation Implications

This study recorded the distribution of koalas in significantly different densities across differing vegetation types, with the highest density occurring in the habitat with the most desirable food species. However, the majority of koalas within this population do not occur in this high quality habitat, as it covers only 5% of the island. Witt and Pahl [56] were first to record significant koala populations within low-quality habitat. Prior to this it was presumed that koalas in Queensland were largely restricted to riverine communities whose predominant vegetation was river red gum (*Eucalyptus camaldulensis*) and coolabah (*Eucalyptus coolabah*) [25]. Recent results from the Mulgalands also suggested a wider variety of vegetation communities utilized by koalas [25]. The failure of management due to underestimation of the koala population on Kangaroo Island [24] highlights the need to stratify population surveys by vegetation types, surveying in a complete range of habitats. While high quality habitats warrant conservation efforts, these results advocate efforts to protect vegetation communities that might traditionally have been considered to have low fauna conservation value, as they may still be essential for maintaining viable, widespread, low-density koala populations [22], [56].

There is general agreement that habitat destruction poses the greatest proximate threat to the conservation of koala populations [11], [57]. Unfortunately, determining which tree species are most preferred by koalas, and therefore should be protected, has been difficult [11]. A complex set of factors has been associated with koala habitat quality. These factors vary widely across regions and include floristic composition, water availability, leaf nutrients, soil type, topography, land use and fire regimes [16], [58], [59]. Given the broad-scale distribution of koalas, investigating differing vegetation communities and the koala densities they support may be a more useful approach to management and conservation planning.

### Urban areas

Urban areas, particularly in southeast Queensland, can support substantial populations of koala [60], [61]. Dique et al. [8] estimated koala density in the urban habitats of the Pine Rivers Shire, Queensland, to be between 0.06 and 0.42 ha^−1^, or 25% of the regional population. Urban areas account for 548 hectares, or 11%, of the total area of Magnetic Island [42].

This study did not assess the density of koalas in the urban areas, as the FSCM would be inappropriate within an urban setting. Transect searches could be difficult due to restricted access on private property, but more importantly, human interference from landscape maintenance and irrigation would result in inaccuracies when using the FSCM. However, multiple sightings of koalas and their signs (pellets and scratches) were seen or reported within urban areas during the study period. Based on anecdotal reports (Petersen pers. comm), it is possible that koala abundance in the urban habitats of Magnetic Island is relatively high. The omission of these areas means that the true island-wide population of koalas is greater than the estimated 825±175 SE reported in this study. In the interest of an estimation of the total population size, if we assume that koalas occur within urban areas on Magnetic Island at the same average density as across the other habitats on the island and the proportional uncertainty remains constant, then we might expect that another 102±21 koalas occur in urban areas, making the total koala population of Magnetic island around 927±195 (SE). Community-response surveys have been used in other regions of Queensland in the past, primarily for determining distribution; however, their accuracy in estimating abundance is controversial [6], [10], [11]. A further survey designed to determine koala population in urban areas would improve the quality of this population estimate.

### Conclusion

The most advantageous time to enact conservation management is before a population has been reduced to a point where opportunities become limited [62]. There appears to be sufficient evidence to conclude that the broad-scale distribution of koalas has decreased by at least 50%, with abundance declining as much as 80% in some areas. However, vigorous populations in diverse locations offer unique possibilities for future conservation [6]. Sixteen koalas were originally introduced to Magnetic Island in 1931–32 as part of an attempt to provide island sanctuaries in response to drastic declines in mainland populations. If recent alarming declines in isolated koala populations in New South Wales [63] and in Queensland [17] are representative of koala population trends across their range, then island populations such as that on Magnetic Island may fulfill their original intent as island sanctuaries. Although there currently is no data on population trends on Magnetic Island, the results from this study provide a baseline to assess future trends in koala distribution, density and abundance. There is little ongoing deforestation on the island, with development limited to the bay areas in this otherwise steep and rocky terrain [42]. Therefore, if declines occur here it is unlikely to be due to broadscale habitat destruction and more likely they are the result of other factors, such as climate change, drought or disease.

Divergence in survey results estimating koala abundance is a key factor in uncertainty regarding conservation status and management, directly retarding conservation efforts for the species. Until robust estimates of abundance can be achieved, inconsistent estimates will continue to hinder conservation efforts [6], [7], [9], [10]. The advantages of using the FSCM to estimate koala abundance include the ability to readily locate koala fecal pellets, as opposed to the more elusive koalas, it is non-invasive [7], [32] and fecal pellets can be used for a variety of additional investigations including genetics and disease analysis [32]. Application of the FSCM across broader areas of the koala's geographic range has the potential to substantially improve our understanding of koala population dynamics.
